# Methodological Design for Three‐Dimensional Assessment of Maxillary and Mandibular Impacted Canines by Cone‐Beam Computed Tomography: A Scoping Review

**DOI:** 10.1111/ocr.70154

**Published:** 2026-07-02

**Authors:** Marcela Gurgel, Ana Carolina Saraiva, Amanda Barbosa Pereira, Francisco Samuel Rodrigues Carvalho, Eduardo Frederico Eduardo Maferano, Aron Aliaga‐Del Castillo, Luis Ernesto Arriola‐Guillén, Cauby Maia Chaves Junior, Lucia Cevidanes, Fabio Wildson Gurgel Costa

**Affiliations:** ^1^ Department of Dental Clinic School of Dentistry, Federal University of Ceará Fortaleza Ceará Brazil; ^2^ Department of Orthodontics and Pediatric Dentistry School of Dentistry, University of British Columbia Vancouver British Columbia Canada; ^3^ Department of Dental Clinic, School of Dentistry Federal University of Ceará Sobral Ceará Brazil; ^4^ Department of Orthodontics and Pediatric Dentistry School of Dentistry, University of Michigan Ann Arbor Michigan USA; ^5^ Division of Orthodontics and Division of Oral and Maxillofacial Radiology School of Dentistry, Universidad Científica del Sur Lima Peru; ^6^ Department of Orthodontics Adams School of Dentistry, University of North Carolina Chapel Hill North Carolina USA

**Keywords:** cone‐beam computed tomography, impacted canine, morphology, root resorption, three‐dimensional analyses

## Abstract

To map and critically synthesize the methodological approaches used for the CBCT‐based evaluation of maxillary and mandibular impacted canines (ICs), with emphasis on imaging protocols, anatomical variables, and diagnostic frameworks. This scoping review followed PRISMA‐ScR guidelines. A three‐step search strategy was applied across PubMed, Embase, Web of Science, Scopus, LIVIVO, Cochrane Library, LILACS, and Google Scholar, using MeSH terms and free‐text keywords related to ICs and CBCT. Data on sample characteristics, CBCT acquisition parameters, anatomical assessment criteria, classification systems, segmentation software, and examiner calibration were extracted and synthesized narratively. Forty‐nine observational studies met the inclusion criteria. Substantial heterogeneity was observed in CBCT equipment, voxel size, field of view, exposure settings, image orientation, segmentation tools, and calibration procedures. Most studies evaluated maxillary canines, whereas mandibular impactions were rarely assessed (6.12%). The most frequently analysed characteristics were the impacted canine's position within the alveolar bone, its involvement in adjacent tooth resorption, and its spatial relationship to surrounding teeth. Major methodological gaps were identified in CBCT acquisition protocols, image orientation, examiner reliability, classification systems, and the assessment of follicular and impacted canine morphology, volume, shape, and anatomical relationships to adjacent structures widely used classification systems, particularly Ericson–Kurol, showed variable adaptations and limited standardization. Most studies evaluated maxillary canines, focusing on bucco‐palatal position, root resorption, and the Ericson and Kurol classification, although CBCT methodologies were inconsistently reported. Standardized acquisition, orientation, and measurement protocols may improve diagnostic consistency and support more reliable orthodontic treatment planning and clinical decision‐making for impacted canines.

## 1 Introduction

Permanent canines play a critical role in functional occlusion and in establishing an aesthetically balanced smile [1]. Canine impaction is defined as the failure of eruption within the expected physiological period despite complete root development [2]. Under normal conditions, maxillary canines erupt between 11 and 13 years of age, with root formation completed by 13–15 years [3], persistence beyond this timeframe indicates impaction.

The prevalence of impacted canines (ICs) ranges from 1% to 3% in the general population and is more frequent in females, with an approximate 2:1 ratio [4]. Clinical consequences include loss of arch space, migration of adjacent teeth, root resorption, and development of dentigerous cysts [4, 5]. In more complex cases, the root apex may lie near the maxillary sinus floor, increasing the risk of anatomical complications [3]. Ectopic positioning has also been associated with nasal septum deviation [6].

Panoramic radiography remains a common first‐line imaging modality; however, its two‐dimensional nature limits spatial accuracy and precludes precise assessment of anatomical relationships [7, 8]. Cone‐beam computed tomography (CBCT), however, provides detailed three‐dimensional (3D) visualization of tooth position, surrounding bone morphology and density, and proximity to adjacent structures, and is now regarded as the imaging modality of choice for evaluating ICs [6].

The prognosis of orthodontic traction depends largely on the 3D position and angulation of the impacted tooth, its distance from the occlusal plane, and the presence and severity of resorption in adjacent roots [9]. Therefore, accurate diagnosis is essential to optimize treatment outcomes and minimize complications such as ankylosis, periodontal damage, and further resorption [5].

Despite agreement on the relevance of CBCT for clinical planning, the literature exhibits substantial methodological heterogeneity, which hinders protocol standardization and limits comparability across studies [7]. To date, no comprehensive synthesis has examined the principal methodological approaches used for 3D assessment of maxillary and mandibular ICs using CBCT.

Accordingly, this scoping review addresses the question: “What methodological designs have been employed for the three‐dimensional assessment of maxillary and mandibular ICs using CBCT?” Synthesizing this evidence will clarify the current state of research, identify methodological gaps and inconsistencies, and support the development of more standardized diagnostic protocols. Ultimately, this may contribute to more accurate diagnosis and improved clinical decision‐making in the management of ICs.

## 2 Materials and Methods

### 2.1 Study Design

This scoping review was performed following the PRISMA‐ScR (Preferred Reporting Items for Systematic Reviews and Meta‐Analyses—Extension for Scoping Reviews) checklist [10]. The protocol was prospectively registered on the Open Science Framework (OSF) to ensure methodological transparency and reproducibility.

### 2.2 Research Question

This review addressed the following question: What methodological designs are used for the three‐dimensional assessment of maxillary and mandibular ICs using cone‐beam computed tomography (CBCT)?

### 2.3 Search Strategy

A comprehensive three‐step search strategy was employed to identify all relevant studies for inclusion in this scoping review:

- In the first step, an exploratory search was conducted in PubMed to identify sentinel studies and to extract Medical Subject Headings (MeSH), relevant keywords, and additional search terms. Both free‐text terms and MeSH descriptors were used to construct the final search algorithm. The free‐text terms included *Canine*, *IC*, *Ectopic Canine*, *Unerupted Canine*, and *Retained Canine*. The primary MeSH descriptor was *Cone‐Beam Computed Tomography*, along with its recognized synonyms. Search terms were combined using the Boolean operator AND, whereas synonyms were combined using OR. The algorithm was adapted to the syntax and indexing of each database and applied consistently across all platforms (Table S1), ensuring reproducibility and comprehensiveness.
- In the second step, the initial systematic search was performed on August 20, 2025, across the following databases: PubMed, Embase, Web of Science, Scopus, LIVIVO, Cochrane Library, LILACS, and Google Scholar (for grey literature).
- In the third step, an updated search was conducted on October 2, 2025, to capture recently published studies prior to data extraction and final synthesis, ensuring that all eligible and up‐to‐date records were included.

### 2.4 Eligibility Criteria

For this scoping review, clinical trials and observational studies that assessed maxillary and mandibular ICs using cone‐beam computed tomography (CBCT) were included. Exclusion criteria comprised cadaver studies, non‐human samples or studies involving non‐canine human teeth, studies employing imaging modalities other than CBCT, studies not written in the Latin alphabet, case reports or case series, narrative reviews, systematic reviews, duplicate publications, diagnostic accuracy or validation studies, concordance studies, and studies whose primary objective was not the three‐dimensional assessment of the morphology or position of ICs.

### 2.5 Study Screening and Selection

After retrieval from all databases, references were exported to EndNote (Thomson Reuters, Philadelphia, PA, USA) for duplicate removal. The deduplicated records were then imported into Rayyan (Qatar Computing Research Institute, Doha, Qatar), where two reviewers independently screened titles and abstracts using the predefined eligibility criteria.

Studies considered potentially eligible were retrieved for full‐text evaluation. During this phase, the inclusion and exclusion criteria were reapplied to determine final eligibility. To minimize selection bias, disagreements between reviewers were resolved through discussion and consensus, with the involvement of a third reviewer, an experienced methodologist in scoping reviews and a subject‐matter expert.

### 2.6 Data Extraction

Data extraction was carried out independently by three independent reviewers. The following information was collected from each included study: authors, year of publication, country, study objectives, study design, sample size, IC location (maxilla or mandible, unilateral or bilateral, right or left side), CBCT acquisition parameters, the relationship of the IC to the midline, the presence of root resorption in adjacent teeth, and the specific teeth affected. The extracted data were organized in a Microsoft Excel spreadsheet. No specialized software was used for data extraction or management.

### 2.7 Data Compilation, Summary, and Presentation

Data compilation focused on identifying and summarizing the methodological designs employed for the three‐dimensional assessment of maxillary and mandibular ICs using CBCT.

Findings were synthesized narratively and presented as relative percentages, enabling the identification of methodological trends, heterogeneity, and gaps within the literature. This approach provided a comprehensive overview of the most frequently applied methods, highlighted inconsistencies across studies, and offered insights to inform future research directions in the field.

## 3 Results

### 3.1 Study Screening and Selection Outcomes

The initial search of the electronic databases identified 5579 studies, in addition to 300 records retrieved from the grey literature. After removing duplicates, a single reviewer independently screened 3023 titles and abstracts from the primary databases and 288 from the grey literature. The remaining 309 studies were assessed in full text by the authors. Critical appraisal at this stage resulted in the exclusion of 260 studies. Therefore, 49 studies were included in the final synthesis (Figure 1).

**FIGURE 1:**
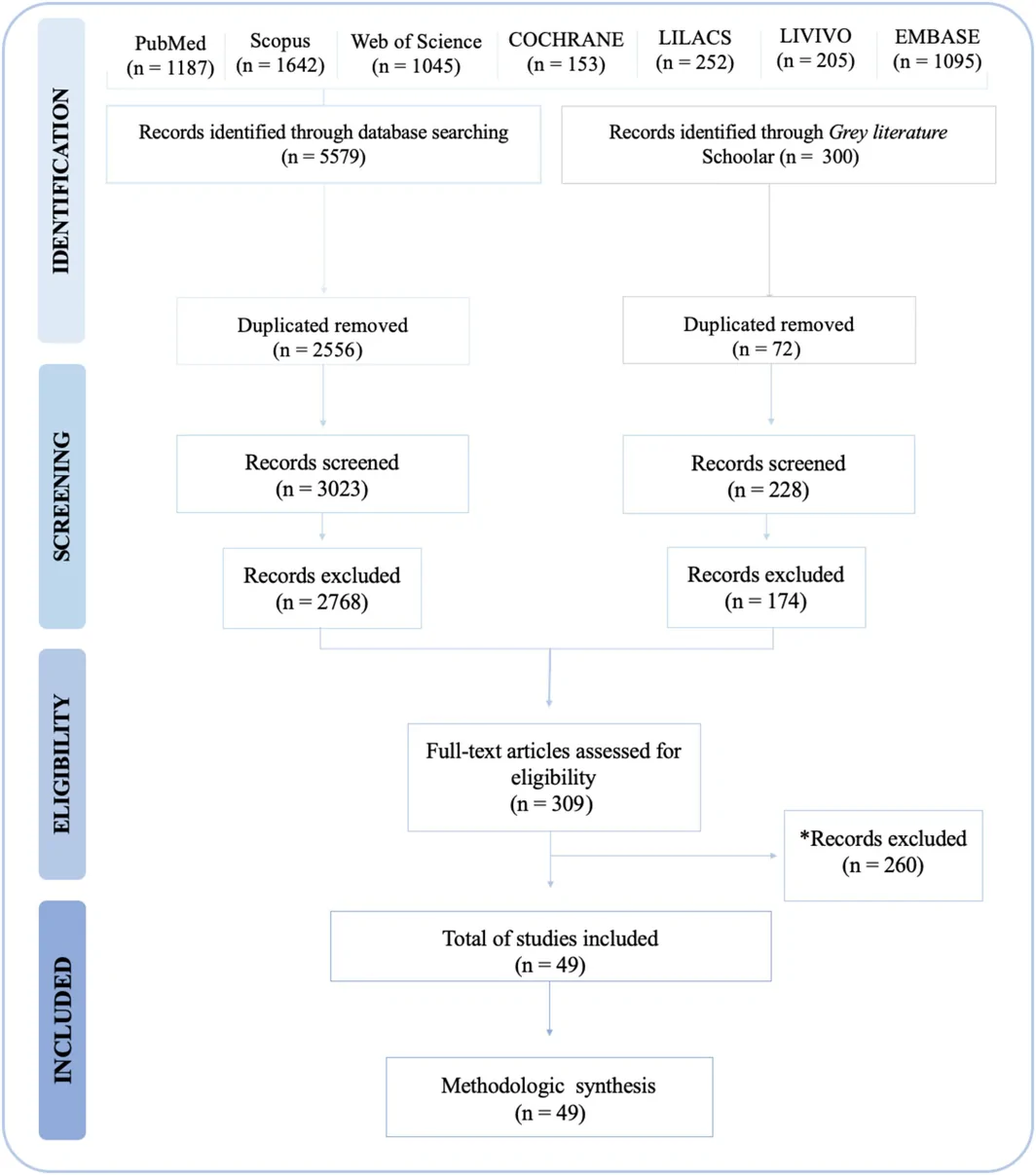
Study screening and selection. The search identified 5879 records (5579 from databases, 300 from grey literature). After duplicates were removed, 3023 database and 288 grey literature records were screened. Full texts of 309 studies were assessed, with 260 excluded, leaving 49 studies for final synthesis.

### 3.2 Study Characteristics

Among the included studies, 26 were conducted in Asia (53.06%) [4, 6, 11, 12, 13, 14, 15, 16, 17, 18, 19, 20, 21, 22, 23, 24, 25, 26, 27, 28, 29, 30, 31]. Most studies adopted a retrospective cross‐sectional design, accounting for 45 studies (91.84%) [4, 6, 11, 12, 13, 14, 15, 16, 17, 18, 19, 20, 21, 22, 23, 24, 25, 26, 27, 28, 29, 30, 31, 32, 33, 34, 35, 36, 37, 38, 39, 40, 41, 42, 43, 44, 45, 46, 47, 48, 49, 50, 51, 52, 53, 54, 55]. Six studies used a case–control design [29, 41, 44, 46, 47, 53].

Across all studies, a total of 5772 patients were evaluated, of whom 60.01% were female. The mean age of participants ranged from 10.9 to 26 years; however, 16 studies did not report age data [13, 17, 20, 23, 27, 28, 31, 32, 33, 34, 39, 41, 44, 47, 48, 51].

A total of 5390 ICs were identified across maxillary and mandibular sites. Forty‐two studies examined only maxillary canines [6, 11, 13, 14, 15, 16, 17, 20, 21, 23, 24, 25, 26, 27, 28, 29, 30, 31, 32, 33, 35, 36, 37, 39, 40, 41, 42, 43, 44, 45, 46, 47, 48, 49, 50, 51, 52, 53, 54, 55, 56, 57], three assessed mandibular canines [19, 34, 38], and four included both arches [4, 12, 18, 25].

Among the 49 included articles, 30 (61.22%) reported evaluating both unilateral and bilateral cases [11, 12, 14, 16, 17, 18, 20, 21, 22, 25, 26, 27, 29, 31, 32, 33, 36, 37, 40, 42, 43, 44, 45, 46, 49, 50, 51, 52, 54, 55, 57, 58], while 13 focused exclusively on unilateral cases [4, 6, 13, 15, 19, 23, 32, 35, 38, 39, 41, 45, 47, 56], and six did not report this information [22, 24, 28, 30, 34, 48, 53]. Regarding the affected side, 19 studies did not specify whether ICs were located on the right or left side [4, 18, 19, 21, 25, 28, 29, 30, 31, 34, 36, 38, 44, 45, 48, 52, 53, 54, 57]. Among the 30 studies reporting laterality, 1326 ICs were identified on the right side and 1279 on the left [6, 11, 12, 13, 14, 15, 16, 17, 20, 22, 23, 24, 27, 30, 31, 33, 35, 37, 39, 40, 41, 42, 43, 46, 47, 49, 50, 51, 55, 56, 58] (Table 1).

**TABLE 1:** Descriptive characteristics of included studies on impacted canine.

Authors	Year	Country	Continent	Study design	Total sample (*n*)	Male (*n*)	Female (*n*)	Mean age (SD)	Impacted canines (*N*)	Maxilla Mandible (*n*)	Uni—U ou Bilateral‐B (*n*)	Impaction in the right—R/left—L side (*n*)
Liu et al.	2008	China	Asia	Retrospective cross‐sectional case–control	175	55	120	16.9 (±6.9)	210	Maxilla (210)	U (140)/B (70)	R‐ NR/L‐ NR
Kau et al.	2009	USA	North America	Retrospective cross‐sectional	NR	NR	NR	NR (±NR)	NR	Maxilla (NR)	NR	R‐ NR/L‐ NR
Takahama et al.	2011	Brazil	South America	Retrospective cross‐sectional	50	12	38	NR (±NR)	71	Maxilla (71)	U (29)/B (21)	R‐ 33/L‐ 38
San Martín et al.	2012	USA	North America	Retrospective cross‐sectional	NR	NR	NR	NR (±NR)	28	Maxilla (28)	NR	R‐ NR/L‐ NR
Jung et al.	2012	Republic of Korea	Asia	Retrospective cross‐sectional	63	28	35	18.4 (±10.6)	73	Maxilla (73)	U (53)/B (10)	R‐ NR/L‐ NR
Kim et al.	2012	South Korea	Asia	Retrospective cross‐sectional	148	59	89	NR (±NR)	186	Maxilla (186)	U (110)/B (76)	R‐ 95/L‐ 91
Oberoi et al.	2012	USA	North America	Retrospective cross‐sectional	29	24%	76%	16.6 (±NR)	42	Maxilla (42)	U (16)/(26)	R‐ NR/L‐ NR
Becker et al.	2013	Israel	Asia	Retrospective case study	14	7	7	NR (±NR)	15	Maxilla (15)	U (13)/B (1)	R‐ 4/L‐ 11
Lai et al.	2013	United Kingdom	Europe	Retrospective cross‐sectional	113	39	74	19.35 (±13.65)	134	Maxilla (134)	U (92)/B (42)	R‐ 64—L‐ 70
Liuk et al.	2013	Austra421049/2025‐1 and 30442/2026‐7lia	Oceania	Retrospective cross‐sectional case–control	40	20	26	13.9 (±NR)	46	Maxilla (46)	NR	R‐ NR/L‐ NR
Almuhtaseb et al.	2014	China	Asia	Retrospective cross‐sectional	46	17	29	16.5 (±6.2)	56	Maxilla (51) Mandible (5)	U (40)/B (8)	R‐ 35/L‐ 21
Santos et al.	2014	Brazil	South America	Retrospective cross‐sectional	79	23	56	22 (±NR)	79	Maxilla (79)	U (51)/B (28)	R‐ 34/L‐ 45
Alqerban et al.	2015	Belgium	Europe	Retrospective cross‐sectional case–control	65	22	43	12.1 (±1.23)	65	Maxila (65)	U (65)	R‐ 33/L‐ 32
da Silva et.	2017	Brazil	South America	Retrospective	16	9	7	11.67 (±NR)	16	Maxilla (16)	U (16)	R‐ NR/L‐ NR
Dağsuyusuyu et al.	2017	Turkey	Europe/Asia	Retrospective cross‐sectional	102	43	59	16.25 (±6.31)	140	Maxilla (140)	NR	R‐ 72/L‐ 68
Dağsuyu et al.	2017	Turkey	Europe/Asia	Retrospective cross‐sectional	102	43	59	16.25 (±6.31)	140	Maxilla (140)	U (64)/B (38)	R‐ 72/L‐ 68
Hettiarachchi et al.	2017	Australia	Oceania	Retrospective cross‐sectional case–control	93	NR	NR	NR (±NR)	59	Maxilla (59)	U (29)/B (30)	R‐ 30/L‐ 29
Zeno/Ghafari	2017	USA	Unites States	Retrospective cross‐sectional	28	8	20	16.06 (±4.9)	38	Maxila (38)	U (18)/B (20)	R‐ NR/L‐ NR
Schubert et al.	2018	Germany	Europe	Retrospective cohort	30	12	18	13.76 (±1.52)	30	Maxilla (30)	U (30)	R‐ 15/L‐ 15
Bertl et al.	2018	Austria	Europe	Retrospective cross‐sectional	88	41	47	25.6 (±NR)	94	Mandible (94)	U (82)/B (6)	R‐ NR/L‐ NR
Leonardi et al.	2018	Italy	Europe	Retrospective cross‐sectional	53	23	30	NR (±NR)	28	Maxilla (28)	U (28)	R‐ 13/L‐ 15
Grisar et al.	2019	Belgium	Europe	Retrospective cross‐sectional	130	48	82	NR (±NR)	162	Maxilla (162)	U (98)/B (64)	R‐ 79/L‐ 83
Kalyani et al.	2019	India	Asia	Retrospective cross‐sectional	24	10	14	NR (±NR)	33	Maxilla (33)	U (15)/B (8)	R‐ 7/L‐ 8
Arriola‐Guillen et al.	2019	Peru	South America	Retrospective cohort	30	11	19	18.16 (±7.32)	45	Maxilla (45)	U (15)/B (30)	R‐ 25/L‐ 20
Alyami et al.	2020	Saudi Arabia	Asia	Retrospective cross‐sectional	107	38	69	20.4 (±11.1)	107	Maxilla (99) Mandible (8)	U/B	R‐ NR/L‐ NR
Muñoz‐Domon et al.	2020	Chile	South America	Retrospective cross‐sectional	135	52	83	12 (±2.26)	181	Maxilla (181)	U (89)/B (46)	R‐ 98/L‐ 83
Yilmaz et al.	2020	Turkey	Asia	Retrospective cross‐sectional	169	96	73	17.91 (±6.52)	169	Maxilla (169)	NR	R‐ NR/L‐ NR
Ali et al.	2021	Iraq	Asia	Retrospective cross‐sectional	41	9	32	20.8 (±11.1)	56	Maxilla (56)	U (26)/B (15)	R‐ 27/L‐ 29
Amuk et al.	2021	Turkey	Europe/Asia	Retrospective longitudinal	38	16	22	15.36 (±1.67)	38	Maxilla (38)	U (38)	R‐ NR/L‐ NR
Karabas et al.	2021	Turkey	Europe/Asia	Retrospective cross‐sectional	58	30	28	26 (±NR)	66	Mandible (66)	U (50)/B (16)	R‐ NR/L‐ NR
Iftikhar et al.	2021	Pakistan	Asia	Retrospective cross‐sectional	43	NR	NR	16.02 (±4.017)	47	Maxilla (NR)/Mandible (NR)	U (NR)/B (NR)	R‐ 23/L‐ 24
Al‐Tawachi et al.	2021	Jordan	Asia	Retrospective cross‐sectional	139	33	106	21.34 (±4.26)	191	Maxilla (191)	U (87)/B (52)	R‐ 94/L‐ 97
Cao et al.	2021	China	Asia	Retrospective cross‐sectional case–control	290	114	176	NR (±NR)	145	Maxilla (145)	U (145)	R‐ NR/L‐ NR
Rodríguez‐Cárdenas et al.	2021	Brazil	South America	Retrospective comparatative cohort	33	16	17	19.5 (±NR)	45	Maxilla (45)	U (NR)/B (NR)	R‐ NR/L‐ NR
Razeghinejad et al.	2022	Iraq	Asia	Retrospective cross‐sectional	47	NR	NR	NR (±NR)	47	Maxilla (47)	U (47)	R‐ 28/L‐ 19
Yang et al.	2022	Korea	Asia	Retrospective cross‐sectional	74	32	42	10.9 (±2.4)	74	Maxilla (87)	U (61)/B (26)	R‐ 45/L‐ 42
Hassam et al.	2022	Iraq	Asia	Retrospective cross‐sectional	2230	798	1432	24.77 (±6.06)	1432	Maxilla (1559) Mandible (671)	U (2230)	R‐ NR/L‐ NR
Mejía‐Milian et al.	2022	Peru, Colombia/Dominican Republic	South America/North America	Retrospective cross‐sectional	35	18	17	14.37 (±10.26)	43	Mandible (43)	U (27)/B (8)	R‐ 21/L‐22
Montes‐Diaz et al.	2022	Spain	Europe	Retrospective cross‐sectional case‐control	100	44	56	NR (±NR)	50	Maxilla (50)	U (50)	R‐ 29/L‐ 21
Ross et al.	2022	USA	North America	Retrospective cross‐sectional	207	73	119	15.28 (±8.56)	314	Maxilla (315)	U (100)/B (214)	R‐ 163/L‐ 151
Viktoraviciute et al.	2023	Lithuania	Europe	Retrospective cross‐sectional split mouth	40	14	26	15.68 (±4.58)	20	Maxilla (40)	U (40)	R‐ 23/L‐ 17
Alshalawi et al.	2023	Saudi Arabia	Asia	Retrospective cross‐sectional	59	20	39	NR (±NR)	83	Maxilla (83)	U (35)/B (48)	R‐ 40/L‐ 43
Faiq/Dewachi	2023	Iraq	Asia	Retrospective cross‐sectional	100	39	61	NR (±NR)	100	Maxilla (100)	U (100)	R‐ 53/L‐ 47
Kucukkaraca et al.	2023	Turkey	Europe/Asia	Retrospective cross‐sectional	46	22	24	14.58 (±NR)	46	Maxilla (46)	U (46)	R‐ 23/L‐ 23
Keener et al.	2023	USA	North America	Retrospective cross‐sectional	83	33	50	16.6 (±5.9)	120	Maxilla (120)	U (NR)/B (NR)	R‐ NR/L‐ NR
Oh et al.	2023	South Korea	Asia	Retrospective cross‐sectional	42	19	23	NR (±NR)	56	Maxilla (56)	U (28)/B (28)	R‐ NR/L‐ NR
Pajevic et al.	2023	Serbia	Europe	Retrospective cross‐sectional	94	31	63	19.83 (±5.18)	116	Maxilla (116)	U (73)/B (21)	R‐ 36/L‐ 37
Alshalawi et al.	2024	Saudi Arabia	Asia	Retrospective cross‐sectional	22	10	12	15.8 (±3.1)	22	Maxilla (22)	U (22)	R‐ 12/L‐ 10
Narmada et al.	2024	Indonesia	Asia	Retrospective cross‐sectional	22	NR	NR	NR (±NR)	32	Maxilla (32)	NR	R‐ NR/L‐ NR

Abbreviations: B, bilateral; L, left; NR, not reported; *R*, right; SD, standart deviation; U, unilateral.

### 3.3 Reliability

Among the included studies, 33 articles reported having performed examiner reliability tests [11, 12, 13, 15, 17, 19, 20, 22, 23, 24, 25, 29, 30, 31, 32, 35, 38, 39, 40, 41, 42, 43, 44, 45, 47, 49, 50, 51, 53, 54, 55, 56, 57, 58]. Of these, six studies reported inter‐examiner reliability [11, 12, 13, 20, 23, 57], while 21 reported intra‐examiner reliability tests [10, 15, 19, 22, 28, 29, 30, 31, 32, 35, 38, 39, 41, 42, 43, 44, 47, 49, 51, 53, 54, 55, 56, 59], and six reported both intra‐ and inter‐examiner calibration [17, 26, 32, 40, 45, 50, 57].

With respect to the specialty field of the evaluators, 10 articles (20.41%) reported that the examiners were not radiologists [4, 6, 11, 22, 24, 39, 42, 49, 56, 57, 58], whereas seven articles (14.29%) indicated the participation of radiology specialists [13, 19, 21, 25, 37, 40, 51]. In contrast, 32 articles (65.31%) did not provide information on the examiners' specialty [12, 14, 15, 16, 17, 18, 20, 22, 23, 24, 27, 28, 29, 30, 31, 32, 33, 34, 35, 36, 38, 41, 42, 43, 44, 45, 46, 48, 50, 52, 53, 54, 55].

Regarding the type of measurements used, 11 articles (22.45%) reported only quantitative measurements [6, 14, 15, 18, 35, 38, 39, 40, 41, 52, 56, 59], three articles (6.12%) reported only qualitative measurements [33, 34, 48], and 35 articles (71.43%) combined both quantitative and qualitative measurements [4, 11, 12, 13, 16, 17, 19, 20, 21, 22, 23, 24, 25, 26, 27, 28, 29, 30, 31, 32, 36, 37, 42, 43, 44, 45, 47, 49, 50, 51, 53, 54, 55, 57]. Only six studies reported the use of Dahlberg's formula [19, 38, 39, 43, 51, 58]. The intraclass correlation coefficient (ICC) was used in 19 studies [11, 13, 15, 17, 19, 20, 29, 32, 35, 40, 41, 44, 45, 52, 55, 56, 57, 58], with values ranging from 0.63 [15, 32] to 0.99 [15, 32, 35, 52]. The kappa coefficient was reported in 12 studies [11, 19, 25, 31, 40, 43, 44, 45, 49, 55, 57, 58], of which two reported values greater than 0.90 [43, 44] and 1 reported a kappa value equal to 1.00 [58].

### 3.4 Acquisition Parameters

Information related to acquisition parameters, CBCT device type, voxel size, field of view (FOV), image orientation, and the software used for measurements and analysis is presented in Table 2.

**TABLE 2:** Characteristic description considering CBCT acquisition variables.

Authors	Year	CBCT machine	Voxel size (mm^3^)	FOV (cm)	Kvp	mA	Image orientation	Software/Version/Open‐access
Liu et al.	2008	NewTom Model QR‐DVT 9000	NR	NR	NR	NR	NR	NewTom 9000/3.1/NO
Kau et al.	2009	Sirona Galileos System	0.15–0.3	16 × 16	NR	NR	NR	Galaxis/NR/NO
Takahamaet al	2011	i‐CAT	NR	6 × 8	NR	NR	YES	ImplantViewer/1.4/NO
San Martín et al.	2012	Sirona Galileos CBCT	NR	NR	NR	NR	NR	Sidexis XG/NR/NO
Jung et al.	2012	DCT Pro	0.4	20 × 19	90	4	NR	Ez3D2009 CBCT software (Vatech Co.)/NR/NR
Kim et al.	2012	SOMATOM Sensation 10	NR	NR	NR	NR	NR	NR/NR/NR
Oberoi et al.	2012	Hitachi MercuRay CBCT	NR	30.5 × 30.5	120	15	YES	Dolphin 3D Imaging (Chatsworth, CA, USA)/10.5/NO
Becker et al.	2013	NR	NR	NR	NR	NR	NR	NR/NR/NR
Lai et al.	2013	3D Accuitomo XYZ Slice View Tomograph, Morita Corp., Kyoto, Japan	0.08	4 × 4/6 × 6/8 × 8	80	5	YES	i‐Dixel, Morita Corp., Kyoto, Japan/NR/NO
Liuk et al.	2013	NR	NR	NR	NR	NR	YES	Dolphin Imaging (Dolphin Imaging & Management Solutions, Chatsworth, Califórnia)/11.0/NO
Almuhtaseb et al.	2014	Kavo 3D CBCT machine	NR	12 × 12	120	5	YES	InVivo Dental Anatomage/5.1.10/NO
Santos et al.	2014	KODAK 3D K9000 CBCT unit	NR	NR	NR	NR	NR	KDIS3D CBCT/2.1.11/NO
Alqerban et al.	2015	3D Accuitomo‐XYZ Slice View Tomograph OR Scanora 3D CBCT	0.125/0.2	3 × 4/7.5 × 10	80/85	10	NR	NR/NR/NR
da Silva et al.	2017	Prexion3D/i‐CAT	0.125/0.2	8 × 8/6 × 6	90/120	4/8	NR	Prexion 3D Viewer software (PreXion Inc., San Mateo, Ca, USA)/NR/NO
Dağsuyu et al.	2017	Promax 3D. Mid	NR	NR	94	14	NR	O&O (Materialize, Leuven, Belgium)/NR/NO
Dağsuyu et al.	2017	Promax 3D Mid machine	NR	NR	94	14	NR	O&O (Materialize, Leuven, Belgium)/NR/NO
Hettiarachchi et al.	2017	Classic i‐CAT/Next Generation i‐CAT	0.2/0.25	NR	NR	NR	YES	In Vivo software (Anatomage, San Jose, Calif)/5.3/NO
Zeno and Ghafari	2017	NR	NR	NR	NR	NR	NR	Ez3D Plus 3D CDViewer software (Vatech Global, Gyeonggi‐do, Korea)/1.2.6.6/NO
Schubert et al.	2018	Master3DS Computerized Tomography System	NR	16 × 10	90	3.5	NR	EzImplant‐CD‐Viewer (INFINITT Healthcare Co. Ltd., Philipsburg, New Jersey, USA)/1.5/NO Ez3D2009‐3D‐CDViewer (Vatech Co. Ltd., Korea)/1.2/NO
Bertl et al.	2018	Philips Tomoscan SR‐6000/Siemens Somatom Sensation 4/3D Accuitomo MCT‐1	NR	10 × 12/NR/NR	120/120/90	75/80/5	NR	Easy Vision workstation, Dental (Philips, Best, Netherlands)/2.1/NO
Leonardi et al.	2018	NewTom 3G	0.25	NR	110	6.19	YES	Mimics (Mimics Research, Materialize NV, Liege, Belgium)/19.0.0.347/NR
Grisar et al.	2019	roMax 3D, 3D Accuitomo 170, or Newtom VGi evo	NR	NR	NR	NR	NR	IMPAX software (Agfa, Mortsel, Belgium)/NR/NO
Kalyani et al.	2019	NR	NR	NR	NR	NR	NR	NR/NR/NR
Arriola‐Guillen et al.	2019	PaX‐Uni 3D	0.125	8 × 8	89	4.7	NR	Dolphin Imaging & Management Solutions, Chatsworth, California/11.7/NO
Alyami et al.	2020	Carestream Dosimetry of the 9500 3D Cone Beam System, Carestream Dental LLC, 3625, Cumber‐ land Blvd. Ste. 700, Atlanta, GA 30339	NR/	NR	NR	NR	NR	NR/NR/NR
Muñoz‐Domon et al.	2020	Planmeca Promax 3D Classic, Illinois, EUA	0.2	8 × 8	60–90	1–14	NA	Planmeca Romexis Viewer (Planmeca OY, Helsinki, Finland)/NR/NO
Yilmaz et al.	2020	Veraviewepocs 3D R100/F40 device	NR	8 × 5/4 × 4	85–90	3–8	NR	i‐Dixel 2.0 software (J. Morita Corporation, Osaka, Japan)/2/NO
Ali et al.	2021	CS 9300 3D	NR	8 × 8	80	10	NR	CS 3D imaging software (Carestream Dental LLC., Atlanta, USA) (NR) (NO)
Amuk et al.	2021	NewTom 5G unit	0.25	12 × 8	NR	NR	NR	NNT Cone Beam 3D Imaging Software (9.1.0) (NO)
Karabas et al.	2021	Scanora 3D‐X CBCT Unit by Soredex	NR	50 × 50/18 × 16.5	60–90	4–10	NR	OnDemand3DTM by Cybermed (California, USA) (NR) (NO)
Iftikhar et al.	2021	CS 9000	0.076	5 × 3	60–90	NR	YES	3D CS 3600/NR/NO
Al‐Tawachi et al.	2021	CS9500 cone beam 3D system	0.2	15 × 9	90	10	NR	CS 3D imaging software (Carestream Dental, Stuttgart, Germany)/3.2.9/NO
Cao et al.	2021	NewTom VG; QR, Verona, Italy	0.3	NR	110	1–20	YES	SimPlant O&O/13.0/NO
Rodríguez‐Cárdenas et al.	2021	PaX‐Uni 3D	NR	8 × 8	89	4.7	YES	ITK SNAP/NR/YES
Razeghinejad et al.	2022	NR	NR	NR	NR	NR	NR	Romexis software/3.8.2/(NO)
Yang et al.	2022	NR	NR	NR	NR	NR	NR	NR/NR/NR
Hassam et al.	2022	CBCT machine, NewTom VG, QR s.r.l.	0.3	15 × 15	110	1–20	NR	NR/NR/NR
Mejía‐Milian et al.	2022	NR	NR	NR	NR	NR	NR	Dolphin Imaging and Management Solutions (Patterson Dental Supply Inc., Chatsworth, CA)/11.9/NR
Montes‐Diaz et al.	2022	Planmeca Promax 3D MID	NR	NR	54–90	1–14	NR	Invivo 6 KaVo (Kavo, Hatfield, PA, EUA)/NR/NO
Ross et al.	2022	Planmeca ProMax 3D	0.075–0.6	5 × 5.5–23 × 26	NR	NR	YES	Invivo6 Anatomage/NR/NO
Viktoraviciute et al.	2023	3D im‐ aging	NR	15 × 13	105	16	NR	White Fox Imaging/4/NO
Alshalawi et al.	2023	3D Accuitomo, J. Morita MFG. Corp. Japan	NR	NR	NR	NR	NR	NR/NR/NR
Faiq and Dewachi	2023	Carestream 8100	0.15	16 × 12	90	2.5	NR	MIMICS/20.0/NO VR Mesh/NR/NO
Kucukkaraca et al.	2023	Promax 3D Mid machine	NR	NR	94	14	NR	Planmeca Romexis 3D Imaging Program (Planmeca Romexis 3D Imaging Software, Helsinki, Finland)/NR/NO
Keener et al.	2023	NR	NR	NR	NR	NR	YES	3D Slicer/4.11/YES
Oh et al.	2023	Pax‐Zenith3D	NR	NR	NR	NR	YES	OnDemand3D (Cybermed Inc., Seoul, South Korea)/NR/NO
Pajevic et al.	2023	Cranex 3D	0.3	61 × 78	60	6.3–12.5	NR	OnDemand3D (Cybermed Inc., Seoul, South Korea)/NR/NO
Alshalawi et al.	2024	Planmeca ProMax 3D Mid CBCT	NR	NR	90/120	NR	YES	OnDemand 3D App software/1.0.10/NO
Narmada et al.	2024	NR	NR	NR	NR	NR	NR	Dolphin Imaging & Management Solutions, Chatsworth, California/11.7/NO

Abbreviations: NO, no; NR, not reported.

### 3.5 Relationship of the Canine to the Midline

Among the included studies, 23 (47%) evaluated the relationship between the long axis of the IC and the midline [4, 6, 11, 12, 13, 15, 16, 19, 20, 22, 24, 29, 32, 36, 40, 43, 45, 46, 47, 50, 51, 52, 53, 57]. In contrast, 26 studies (53%) did not assess this relationship [4, 6, 11, 12, 13, 15, 16, 19, 20, 22, 24, 29, 32, 36, 40, 43, 45, 46, 47, 50, 51, 52, 53, 57] (Table 3).

**TABLE 3:** Characteristic description considering diagnostic factors related to impacted canine position and tooth resorption.

Authors	Year	Canine long axis to the midline	Tooth resorption evaluation	Affected teeth (*n*)	Central incisor resorption (*n*)	Lateral incisor resorption (*n*)	First premolar resorption (*n*)	Second premolar resorption (*n*)	First molar resorption (*n*)
Liu et al.	2008	YES	YES	99	46	53	NR	NR	NR
Kau et al.	2009	NO	NR	NR	NR	NR	NR	NR	NR
Takahama et al.	2011	YES	YES	22	5	17	0	0	0
San Martín et al.	2012	NO	NA	NA	NA	NA	NA	NA	NA
Jung et al.	2012	NO	YES	22	NR	NR	NR	NR	NA
Kim et al.	2012	NO	YES	92	NR	NR	NR	NR	NR
Oberoi et al.	2012	NO	YES	26	1	25	NR	NR	NR
Becker et al.	2013	YES	YES	16	5	11	NR	NR	NA
Lai et al.	2013	NO	YES	48	7	34	6	1	NA
Liuk et al.	2013	YES	YES	NR	NR	NR	NR	NR	NR
Almuhtaseb et al.	2014	YES	NA	NA	NA	NA	NA	NA	NA
Santos et al.	2014	NO	YES	71	19	48	4	NR	NA
Alqerban et al.	2015	YES	NO	NA	NA	NA	NA	NA	NA
da Silva et.	2017	NO	NO	NA	NA	NA	NA	NA	NA
Dağsuyusuyu et al.	2017	NO	YES	77	NA	77	NA	NA	NA
Dağsuyu et al.	2017	YES	YES	111	NA	77	32	NA	NA
Hettiarachchi et al.	2017	YES	NA	NA	NA	NA	NA	NA	NA
Zeno and Ghafari	2017	YES	NO	NA	NA	NA	NA	NA	NA
Schubert et al.	2018	NO	NA	NA	NA	NA	NA	NA	NA
Leonardi et al.	2018	NO	NO	NA	NA	NA	NA	NA	NA
Bertl et al.	2018	YES	YES	9	2	5	2	0	0
Grisar et al.	2019	NO	NA	NA	NA	NA	NA	NA	NA
Kalyani et al.	2019	NO	NO	NA	NA	NA	NA	NA	NA
Arriola‐Guillen et al.	2019	YES	YES	18	NR	NR	NR	NR	NR
Alyami et al.	2020	NO	NO	NA	NA	NA	NA	NA	NA
Muñoz‐Domon et al.	2020	NO	YES	65	8	50	NA	NA	6
Yilmaz et al.	2020	NO	YES	169	NR	NR	NR	NR	NR
Ali et al.	2021	YES	YES	32	9	21	1	1	NA
Amuk et al.	2021	YES	YES	1	0	1	0	NA	NA
Karabas et al.	2021	NO	YES	12	NR	NR	NR	NR	NR
Iftikhar et al.	2021	NO	NO	NA	NA	NA	NA	NA	NA
Al‐Tawachi et al.	2021	YES	NO	NO	NA	NA	NA	NA	NA
Cao et al.	2021	NO	NO	NA	NA	NA	NA	NA	NA
Rodríguez‐Cárdenas et al.	2021	YES	NO	NA	NA	NA	NA	NA	NA
Razeghinejad et al.	2022	YES	YES	NR	NA	NR	NA	NA	NA
Yang et al.	2022	NO	YES	20	NR	NR	NR	NR	NR
Hassam et al.	2022	YES	YES	31	NR	NR	NR	NR	NR
Mejía‐Milian et al.	2022	YES	YES	15	7	8	NA	NA	NA
Montes‐Diaz et al.	2022	NO	NO	NA	NA	NA	NA	NA	NA
Ross et al.	2022	YES	NO	NA	NA	NA	NA	NA	NA
Viktoraviciute et al.	2023	NO	NA	NA	NA	NA	NA	NA	NA
Alshalawi et al.	2023	NO	NO	NA	NA	NA	NA	NA	NA
Faiq and Dewachi	2023	NO	NO	NO	NO	NO	NO	NO	NO
Kucukkaraca et al.	2023	YES	NO	NA	NA	NA	NA	NA	NA
Keener et al.	2023	YES	YES	234	68	139	27	NA	NA
Oh et al.	2023	YES	NO	NA	NA	NA	NA	NA	NA
Pajevic et	2023	NR	YES	27	17	4	6	NR	NR
Alshalawi et al.	2024	YES	YES	NR	NR	NR	NR	NR	NR
Narmada et al.	2024	NO	YES	20	6	14	NA	NA	NA

Abbreviations: NA, not applied; NR, not reported.

### 3.6 Relationship of the Canine to Adjacent Teeth

Of the 49 included studies, 26 (53.1%) assessed root resorption of adjacent teeth [4, 11, 13, 14, 15, 19, 20, 21, 22, 24, 25, 28, 29, 30, 31, 32, 36, 37, 40, 42, 45, 49, 51, 53, 54, 55, 58]. Of these, 22 studies (84.6%) reported the number of affected teeth, totalling 1015 compromised teeth [4, 11, 14, 19, 20, 21, 22, 24, 25, 28, 29, 30, 36, 37, 40, 42, 45, 49, 51, 55, 58].

Lateral incisors were the most frequently affected teeth, representing 659 cases (64.9%) and reported in 17 studies (65.4%) [11, 19, 20, 22, 24, 27, 28, 29, 30, 36, 37, 40, 42, 45, 49, 51, 54, 55]. Central incisors were the second most affected group, comprising 203 cases (20%) and reported in 14 studies (53.8%) [11, 20, 28, 29, 36, 37, 40, 42, 45, 49, 51, 54, 55].

Less frequently affected teeth included the first premolar (*n* = 78, 7.7%), reported in seven studies (27%), and the second premolar (*n* = 2, 0.2%), reported in three studies (11.5%) [11, 49]. The first molar was involved in six cases (0.6%), reported in two studies (7.7%) [36, 42]. Additionally, 67 cases (6.6%) corresponded to an unspecified group of affected teeth [4, 13, 14, 15, 21, 25, 31, 32, 53, 58] (Table 3).

### 3.7 Position of the IC in the Alveolar Bone

Of the 49 studies included in this review, 43 (87.76%) evaluated the position of the IC within the alveolar bone, predominantly in the bucco‐palatal dimension [6, 11, 13, 14, 15, 16, 17, 18, 19, 20, 21, 22, 23, 24, 25, 26, 27, 28, 30, 31, 32, 33, 35, 36, 37, 38, 39, 40, 41, 42, 43, 44, 45, 46, 47, 49, 50, 51, 52, 53, 54, 55, 57]. Among these, 25 studies (51.02%) described bicortical positioning of ICs [10, 15, 17, 20, 21, 22, 23, 24, 26, 27, 28, 32, 33, 35, 36, 37, 38, 40, 44, 45, 46, 49, 50, 51, 55, 58]. Five studies (10.20%) assessed vertical and horizontal positioning [4, 6, 15, 32, 33, 46], while three studies (6.12%) evaluated mesiodistal orientation [6, 33, 46] (Table 4).

**TABLE 4:** Radiographic evaluation parameters for impacted canines.

	Total/Percentage	Autors			
Impacted canine position within the bone					
Buccal or Palatal	43/87.76%	Ali et al., 2021 Grisar et al., 2019 Razeghinejad et al., 2022 Viktoraviciute et al., 2023 Yang et al., 2022 Alshalawi et al., 2024 Alshalawi et al., 2023 Alyami et al., 2020 Amuk et al., 2021 Becker et al., 2013 Bertl et al., 2018 Karabas et al., 2021	Iftikhar et al., 2021 Jung et al., 2012 Kalyani et al., 2019 Kucukkaraca et al., 2023 Leonardi et al., 2018 Mejía‐Milian et al., 2022 Montes‐Diaz et al., 2022 Munoz‐Domon et al., 2020 Narmada et al., 2024 Al‐Tawachi et al., 2021 Cao et al., 2021 Keener et al., 2023	Alqerban et al., 2015 Hettiarachchi et al., 2017 Lai et al., 2013 Rodrígues‐Cárdenas et al., 2021 Ross et al., 2022 Takahama et al., 2011 Yilmaz et al., 2020 Zeno and Ghafari, 2017 Kim et al., 2012 Arriola‐Guillen et al., 2019 Liuk et al., 2013 Oberoi et al., 2012	Santos et al., 2014 Da Silva et al., 2017 Dağsuyu et al., 2017 Dağsuyu et al., 2017 Faiq and Dewachi, 2023 Oh et al., 2023 Pajević et al., 2023
Bicortical	25/51.02%	Ali et al., 2021 Grisar et al., 2019 Viktoraviciute et al., 2023 Alshalawi et al., 2024 Alshalawi et al., 2023 Becker et al., 2013 Bertl et al., 2018 Karabas et al., 2021 Santos et al.,2014 Da Silva et al., 2017	Dağsuyu et al., 2017 Dağsuyu et al., 2017 Faiq and Dewachi, 2023 Jung et al., 2012 Kalyani et al., 2019 Mejía‐Milian et al., 2022 Narmada et al., 2024 Cao et al., 2021 Keener et al., 2023 Alqerban et al., 2015	Lai et al., 2013 Ross et al., 2022 Takahama et al., 2011 Arriola‐Guillen et al., 2019 Pajević et al., 2023	
Vertical or Horizontal	5/10.2%	Grisar et al., 2019 Alshalawi et al., 2024 Hassam et al., 2022 Kucukkaraca et al., 2023 Alqerban et al., 2015			
Mesial or Distal	3/6.12%	Grisar et al., 2019 Kucukkaraca et al., 2023 Alqerban et al., 2015			
Impacted canine vs Adjacent teeth and structures					
Vertical position to adjacent teeth	7/14.30%	Ali et al., 2021 Grisar et al., 2014 Bertl ela al, 2018 Cao et al., 2021 Ross et al., 2022 Ylmaz el al, 2020 Pajević et al., 2023			
Horizontal position to adjacent teeth	4/8.2%	Ali et al., 2021 Grisar et al., 2014 Cao et al., 2021 Pajević et al., 2023			
Overlap to adjacent incisors	4/8.2%	Becker et al., 2013 Keener et al., 2023 Razeghinejad et al., 2022 Kalyani et al., 2019			
Contact to adjacent teeth	10/20.4%	Amuk et al., 2021 Becker et al., 2013 Bertl et al., 2018 Kalyani et al., 2019 Kucukkaraca et al., 2023 Mejía‐Milian et al., 2022 Aqerban et al., 2015 Lai et al., 2013 Liu et al., 2008 Pajević et al., 2023			
Relation with nasal cavity	2/4%	Amuk et al., 2021 Alshalawi et al., 2023			
Relation with maxillary sinus	1/2%	Amuk et al., 2021			
Relation with mandibular cortical bone	1/2%	Karabas et al., 2021			
Relation with mental foramen	1/2%	Karabas et al., 2021			
Relation with mandibular incisive canal	1/2%	Karabas et al., 2021			
Root resorption adjacent teeth	24/48.97%	Ali et al., 2021 Razeghinejad et al., 2022 Yang et al., 2022 Alshalawi et al., 2024 Amuk et al., 2021 Becker et al., 2013 Bertl et al., 2018 Karabas et al., 2021 Santos et al., 2014 Dağsuyu et al., 2017	Mejía‐Milian et al., 2022 Muñoz‐Domon et al., 2020 Narmada et al., 2024 Keener et al., 2023 Lai et al., 2013 Liuk et al., 2013 Oberoi et al., 2014 Pajević et al., 2023 Rodrígues‐Cárdenas et al., 2021 Yilmaz et al., 2020	Arriola‐Guillén et al., 2019 Dağsuyu et al., 2017 Hassam et al., 2022 Jung et al., 2012	
Impacted canine linear measurement					
Crown width (MD)	4/8.16%	Viktoraviciute et al., 2023 Alshalawi et al., 2023 Alqerban et al., 2015 Pajević et al., 2023			
Crown width (BL)	2/4%	Alshalawi et al., 2023 Alqerban et al., 2015			
Crown length	2/4%	Viktoraviciute et al., 2023 Hettiarachchi et al., 2017			
Root length	7/14.28%	Viktoraviciute et al., 2023 Da Silva et al., 2017 Leonardi et al., 2018 Cao et al., 2021 Arriola‐Guillen et al., 2019 Hettiarachchi et al., 2017 Ylmaz et al., 2020			
Dilaceration length	1/2%	Viktoraviciute et al., 2023			
Overall length	3/6.12%	Amuk et al., 2021 Mejía‐Milian et al., 2022 Montes‐Diaz et al., 2022			
Cusp tip to midline	14/28.57%	Ali et al., 2021 Razeghinejad et al., 2022 Alshalawi et al., 2024 Bertl et al., 2018 Karabas et al., 2021 Dağsuyu et al., 2017 Al‐Tawachi et al., 2021 Keener et al., 2023 Alqerban et al., 2015 Lai et al., 2013	Liu et al., 2008 Takahama et al., 2011 Zeno and Ghafari, 2017 Pajević et al., 2023		
Cusp tip to occlusal plane	14/28.57%	Ali et al., 2021 Razeghinejad et al., 2022 Almuhtaseb et al., 2014 Schubert et al., 2018 Alshalawi et al., 2024 Amuk et al., 2021 Dağsuyu ˘suyu et al., 2017 Mejía‐Milian et al., 2022 Keener et al., 2023 Alqerban et al., 2015	Arriola‐Guillen et al., 2019 Liuk et al., 2013 Oh et al., 2023 Pajević et al., 2023		
Crown length	2/4%	Alshalawi et al., 2023 Zeno and Ghafari, 2017			
Cusp tip to frontal plane	2/4%	Almuhtaseb et al., 2014			
Cusp tip to adjacent teeth	2/4%	Zeno and Ghafari, 2017			
Root apex to midline	4/8.16%	Dağsuyu et al., 2017 Zeno and Ghafari, 2017 Oh et al., 2023 Pajević et al., 2023			
Root apex to occlusal plane	4/8.16%	Almuhtaseb et al., 2014 Dağsuyu et al., 2017 Kau et al., 2009 Pajević et al., 2023			
Impacted canine follicle analysis					
Shape	4/8.16%	Bertl et al., 2018 Santos et al., 2014 Lai et al., 2013 Liu et al., 2008			
With	4/8.16%	Bertl et al., 2018 Santos et al., 2014 Lai et al., 2013 Liu et al., 2008			
Size	4/8.16%	Bertl et al., 2018 Santos et al., 2014 Lai et al., 2013 Liu et al., 2008			
Impacted canine classification systems					
Mirabella and Artun	1/2%	Amuk et al., 2021			
Ericson and Kurol	24/46.93%	Ali et al., 2021 Alshalawi et al., 2024 Bertl et al., 2018 Santos et al., 2014 Dağsuyu et al., 2017 Dağsuyu et al., 2017 Kalyani et al., 2019 Kucukkaraca et al., 2023 Muñoz‐Domon et al., 2020 Narmada et al., 2024	Keener et al., 2023 Lai et al., 2013 Arriola‐Guillen et al., 2019 Oberoi et al., 2012 Almuhtaseb et al., 2014 Amuk et al., 2021 Bertl et al., 2018 Karabas et al., 2021 Kalyani et al., 2019 Mejía‐Milian et al., 2022	Rodríguez‐Cárdenas, 2021 Arriola‐Guillen et al., 2019 Oh et al., 2023	
Stivaros and Mandall	1/2%	Becker et al., 2013			
KPG index	5/10.2%	San Martín et al., 2012 Iftikhar et al., 2021 Hettiarachchi et al., 2017 Kau et al., 2009 Pajević et al., 2023			
Lindauer method	2/4%	Razeghinejad et al., 2022 Kau et al., 2009			
Heithersay's classification	1/2%	Becker et al., 2013			
Mupparapu et al., classification	3/6.12%	Bertl et al., 2018 Karabas et al., 2021 Hassam et al., 2022			
Bertl et al., classification	1/2%	Karabas et al., 2021			
Alessandri et al.	1/2%	Jung et al., 2012			

### 3.8 Relationship of the IC to Adjacent Anatomical Structures

Regarding the relationship between ICs and adjacent anatomical structures, seven studies (14.30%) analysed vertical positioning relative to neighbouring teeth [11, 30, 33, 36, 44, 50, 55]. Four studies (8.2%) evaluated the horizontal position of the IC [11, 33, 44, 55]. Another four studies (8.20%) examined canine overlap with adjacent incisors [13, 20, 27, 45].

Additionally, 10 studies (20.40%) reported direct contact between the IC and neighbouring teeth [6, 19, 20, 27, 29, 36, 41, 46, 49, 55] (Table 4).

### 3.9 Linear Measurements

A total of 14 studies (28.57%) measured the distance from the canine cusp tip to the midline and to the occlusal plane [11, 12, 13, 15, 19, 22, 24, 32, 40, 45, 46, 53, 55, 56, 57, 58]. Root length was evaluated in seven studies (14.28%) [30, 35, 38, 39, 44, 47, 58]. Crown width (mesiodistal) was measured in four studies (8.16%) [16, 35, 46, 55], and the distance from the root apex to the midline was assessed in four studies (8.16%) [22, 31, 52, 55]. Likewise, the distance from the root apex to the occlusal plane was measured in four studies (8.16%) [12, 22, 48, 55].

Other measurements included: Total tooth length in three studies (6.12%) [19, 32, 40, 41]. Bucco‐lingual crown width, crown length, and distance from the cusp tip to the palatal plane in two studies each (4.0%) [16, 35, 46, 47, 52]. Dilatation length, distance from the cusp tip to the frontal plane, and distance from the cusp tip to adjacent teeth, each evaluated in a single study (2.0%) [12, 35, 52] (Table 4).

### 3.10 Follicular Morphology and Classification Systems

Follicular morphology of ICs (including follicle shape, width, and size) was described in four studies (8.16%) [29, 36, 37, 49]. Regarding classification systems, the most frequently used method was that of Ericson and Kurol, applied in 23 studies (46.93%) [6, 11, 12, 15, 19, 21, 22, 27, 28, 32, 36, 37, 39, 42, 45, 49, 54, 57, 58]. The KPG index was the second most frequently used system, identified in five studies (10.20%) [24, 34, 47, 48, 55]. The Mupparapu classification was applied in three studies (6.12%) [4, 21, 36], followed by the Lindauer method in two studies (4.0%) [13, 48]. Other systems—including those proposed by Mirabella and Artun, Stivaros and Mandall, Heithersay, Bertl et al., and Alessandri et al.—were each used in only one study (2.0%) [19, 20, 21, 25].

## 4 Discussion

The present scoping review, to our knowledge, is the first to systematically map the methodological landscape of CBCT‐based three‐dimensional assessment of maxillary and mandibular impacted canines. While previous studies have acknowledged variability in acquisition protocols and classification systems, these observations have remained fragmented and secondary to clinically oriented investigations. In contrast, this review consolidates acquisition parameters, orientation procedures, segmentation strategies, examiner calibration, measurement approaches, anatomical relationship assessments, and classification frameworks into a structured methodological synthesis. By organizing these domains and quantifying their reporting patterns, this study moves beyond descriptive heterogeneity and clarifies where methodological inconsistencies are most concentrated, thereby identifying priority areas for future research standardization.

A total of 49 observational studies were included, demonstrating substantial heterogeneity across assessment protocols. The studies varied in their evaluation of canine position within the alveolar bone, relationships with adjacent teeth and anatomical structures, linear and angular measurements, and follicular morphology (shape, size, and width). Additional variability was observed in the classification systems used, CBCT equipment and acquisition parameters, segmentation and measurement software, reliability procedures, image orientation protocols, and overall three‐dimensional assessment criteria. Collectively, these discrepancies highlight the absence of consensus and the need for unified methodological frameworks for CBCT‐based evaluation of ICs.

Most of the included studies originated from Asia, likely reflecting the high concentration of orthodontic journals and the region's substantial research output in orthodontics. A bibliometric analysis by Almotairy [60] reported a dominant contribution of Asian countries to orthodontic literature, which aligns with the predominance of studies from nations such as Saudi Arabia and Iraq in the present review. The sex distribution identified in the included studies also mirrors established epidemiological patterns, with a higher prevalence of canine impaction in females (60.01%) compared with males (39.99%) [11, 12, 13, 33].

Most studies focused on maxillary ICs, consistent with evidence indicating that mandibular canine impaction is a rare eruptive anomaly [21, 40]. When present, mandibular impactions exhibit a greater tendency for transmigration compared with maxillary canines, often resulting in more severe clinical consequences [36]. In this review, only a small proportion of studies evaluated mandibular canines exclusively, representing 6.12% of all included articles.

Intra‐ and inter‐examiner calibration is essential in studies involving three‐dimensional measurements to enhance methodological rigour, minimize bias, and increase the reliability and external validity of findings [61]. Notably, 32.64% of the included studies did not report any reliability procedures, potentially compromising the consistency and robustness of their measurements. Such findings emphasize the need for rigorous examiner calibration in CBCT‐based assessments of ICs.

Five CBCT scanners predominated across the studies—Planmeca ProMax, NewTom, Carestream, 3D Accuitomo, and i‐CAT—while other models appeared sporadically. Scanner variability, combined with the lack of equipment reporting in approximately 20% of the studies, reduces comparability. Each manufacturer employs proprietary X‐ray tube specifications, detector designs, reconstruction algorithms, and post‐processing techniques, all of which influence image formation [62]. Accordingly, Mackin et al. [63] demonstrated that radiomic features, including density, texture, and morphology, vary across CT scanners even under similar acquisition protocols, underscoring the need for equipment standardization.

Lack of rigour was also evident in the report of CBCT acquisition parameters, particularly voxel size and field of view (FOV). Only 34.4% of the studies reported voxel size, and 49% reported FOV. Such underreporting limits comparability, while the voxel size influences diagnostic performance; the FOV directly affects image quality [64]. Moreover, smaller voxels enhance diagnostic accuracy for root resorption [65], although Liedke et al. [66] found no significant differences when voxel size remained between 0.2 and 0.4 mm, suggesting that variations within this range do not compromise accuracy and that other protocol factors may exert greater influence.

Exposure parameters also showed marked variability: 57% of the studies reported kilovoltage and 47% reported milliampere settings, with values ranging widely (kVp: 54–120, mA: 2.5–80). These parameters significantly influence both image quality [64] and radiation dose [67]. Generally, higher mA—and, to a lesser extent, higher kVp—improve image quality, but settings must align with diagnostic needs [68] and comply with the ALARA principle, as recommended by the International Commission on Radiation Protection.

Only 28.5% of the studies described image reorientation. This step, which involves aligning the dataset to a standardized reference system [69], is essential for reproducibility given the broad heterogeneity in acquisition protocols [70]. Omission of this step compromises measurement reliability and the accuracy of three‐dimensional analyses [71].

In 83.6% of the studies, the software used for tomographic measurements was reported, and the most reported platforms used were Dolphin, Invivo, OnDemand 3D, and Planmeca Romexis Viewer. Software choice is a relevant methodological factor because different platforms may generate distinct texture characteristics, limiting cross‐study comparability [72].

Assessment of the relationship between the IC and the midline—particularly the angulation between the canine long axis and the midline—remains clinically relevant for diagnosis and treatment planning [73]. Power et al. [74] identified angulations ≥ 31° as unfavourable for spontaneous eruption following the extraction of the deciduous canine. Conversely, Alqerban et al. [46] reported that this angle alone is insufficient for prognosis, noting that the angle between the canine and the lateral incisor, as well as the position of the canine cusp relative to the occlusal plane, shows stronger predictive value. In the present review, fewer than half of the studies (47%) assessed this parameter, limiting cross‐study consistency.

The aetiology of dental root resorption of adjacent teeth remains incompletely understood. Several authors propose that resorption results from physical pressure and osteoclastic stimulation induced by the eruption path or by contact between impacted and adjacent teeth [4, 22, 24, 25, 28, 30, 40, 42, 45, 55, 57]. Accordingly, the position of the IC is a critical determinant of resorption severity, particularly in incisors [54].

In this review, 20.40% of studies evaluated direct contact between ICs and adjacent teeth, and 48.98% assessed the presence of root resorption. Narmada et al. [28] reported that increasing impaction severity corresponded to greater incisor resorption. Oberoi et al. [54] classified adjacent tooth resorption and found that 49.6% of affected teeth exhibited resorption, predominantly mild (35.7%). Bertl et al. [36] observed that 74.4% of ICs were in contact with adjacent teeth and that resorption occurred at a considerable frequency. Muñoz‐Domón et al. [42] reported resorption in 35.9% of cases, with the apical third most commonly affected (60%), and severe resorption or pulp involvement occurred in 40%. In addition, the present review indicates that the lateral and central incisors are the most frequently affected teeth by ICs, followed by the first and second premolars. This finding is in accordance with Mitsea et al. [75]. Although prevalence rates varied across studies, the overall evidence consistently indicates a strong association between ICs and adjacent root resorption.

Assessing the alveolar position of ICs is critical for understanding their relationship with adjacent structures and guiding clinical and surgical planning [44]. In particular, evaluation of bucco‐palatal positioning supports estimation of the risk of root resorption in neighbouring teeth and informs the degree of surgical difficulty [22, 58]. In this review, 87.76% of included studies assessed buccal or palatal positioning. While Razeghinejad et al. [13] found no significant association between buccal/palatal impaction and adjacent root resorption, da Silva et al. [38] reported greater lateral incisor resorption in cases of palatal impaction. The relationship between ICs and the cortical plates is essential for predicting the risk of fenestration of adjacent teeth and understanding bone support surrounding the impacted tooth [23, 25, 26, 27]. Keener et al. [45] showed that bicortically positioned canines exhibited the greatest severity of resorption, with central incisor involvement in 55.6% of cases. By comparison, buccal and palatal impactions demonstrated central incisor resorption in 34.5% and 34% of cases, respectively. Despite the clinical relevance of this parameter, only 51% of the studies assessed bicortical positioning.

Evaluation of the vertical and horizontal orientation of the IC is similarly important, as these factors may reflect developmental anomalies and predict interference with the eruption or structural integrity of incisors and premolars [22, 23, 24, 25, 26, 27, 28, 40, 59]. Grisar et al. [33] observed an increased frequency of dental ankylosis in canines positioned higher vertically, above the cementoenamel junction. Conversely, root dilaceration was more frequently associated with horizontally oriented or mesially angulated canines, particularly when positioned palatally [33]. Despite its diagnostic value, only five studies assessed the vertical or horizontal orientation of ICs, revealing an evident gap in the literature. Mesiodistal positioning also warrants consideration, as a more mesially located ectopic canine may compromise the roots of adjacent teeth, including the central incisors, resulting in resorption [11]. In this review, only 6.12% of studies evaluated the mesiodistal relationship of ICs, indicating limited attention to this clinically significant parameter.

Understanding the spatial relationship between ICs and adjacent anatomical structures is crucial for identifying morphological variations and assessing proximity to critical regions [55]. For maxillary canines, the nasal cavity and maxillary sinus are of particular concern during surgical exposure and orthodontic traction [19]. Potential complications include nasal floor perforation, oroantral communication, iatrogenic sinusitis, and chronic pain. However, only two studies examined the relationship between ICs and the nasal cavity, and only one evaluated their proximity to the maxillary sinus, highlighting a substantial gap in anatomical assessments within the current literature. Moreover, Karabas [21] was also the only author to assess the relationship between ICs and the mental foramen or incisive canal. Communication with the mental foramen occurred in 17.10% of transmigrated and 9.70% of ICs, while proximity to the mandibular incisive canal was even more frequent, affecting 60% of transmigrated and 64.5% of ICs. These findings underscore the clinical importance of assessing such anatomical relationships, as direct contact or close approximation may lead to nerve injury, paresthesia, pain, or neuropathic sensory alterations [21].

Assessment of the dental follicle was performed in only 8.16% of studies, representing a limited body of evidence. All four studies evaluated follicular presence, shape, and size. The dental follicle plays a key role in tooth eruption and is influenced by metabolic, cellular, and environmental factors, as well as hormones and growth‐related signals [22, 24]. Some studies reported no association between follicle size or morphology and root resorption, consistent with Bertl et al. [36]. Others indicated that variations in follicle size may correlate with incisor resorption [22, 24]. Santos et al. [37] considered a follicular width ≤ 2 mm as normal and > 2 mm as enlarged, identifying enlargement in 40.82% of cases. However, follicular measurements lack standardization across studies, and no validated protocols currently ensure reproducible assessment. Although Santos et al. [37] found a significant association between follicular width and root resorption, resorption also occurred independently of follicle size.

Several classification systems, such as Ericson and Kurol [76], Lindauer method [59], KPG index [34] (Table 4), have been proposed to determine the position and severity of canine impaction, each based on distinct imaging and morphological criteria. The Ericson and Kurol system was the most frequently applied (46.93%). However, this system of classification still relies on 2D panoramic views and provides parameters for maxillary canine evaluations only. Importantly, the absence of consensus on a universal classification system remains a persistent methodological limitation, compromising standardization and reproducibility.

In the present study, the predominance of retrospective cross‐sectional designs restricts the overall level of evidence and limits the ability to draw causal inferences. In addition, substantial heterogeneity across CBCT equipment, voxel sizes, fields of view, exposure parameters, differences in measure systems, image‐processing, and software limits comparability and precludes quantitative synthesis.

This scoping review provides a comprehensive mapping of methodological approaches used for the three‐dimensional evaluation of maxillary and mandibular ICs, synthesizing evidence from 49 observational studies (Figure 2). The characteristics most often analysed were the impacted canine's position within the alveolar bone, its involvement in adjacent tooth resorption, and its spatial relationship to the surrounding teeth.

**FIGURE 2:**
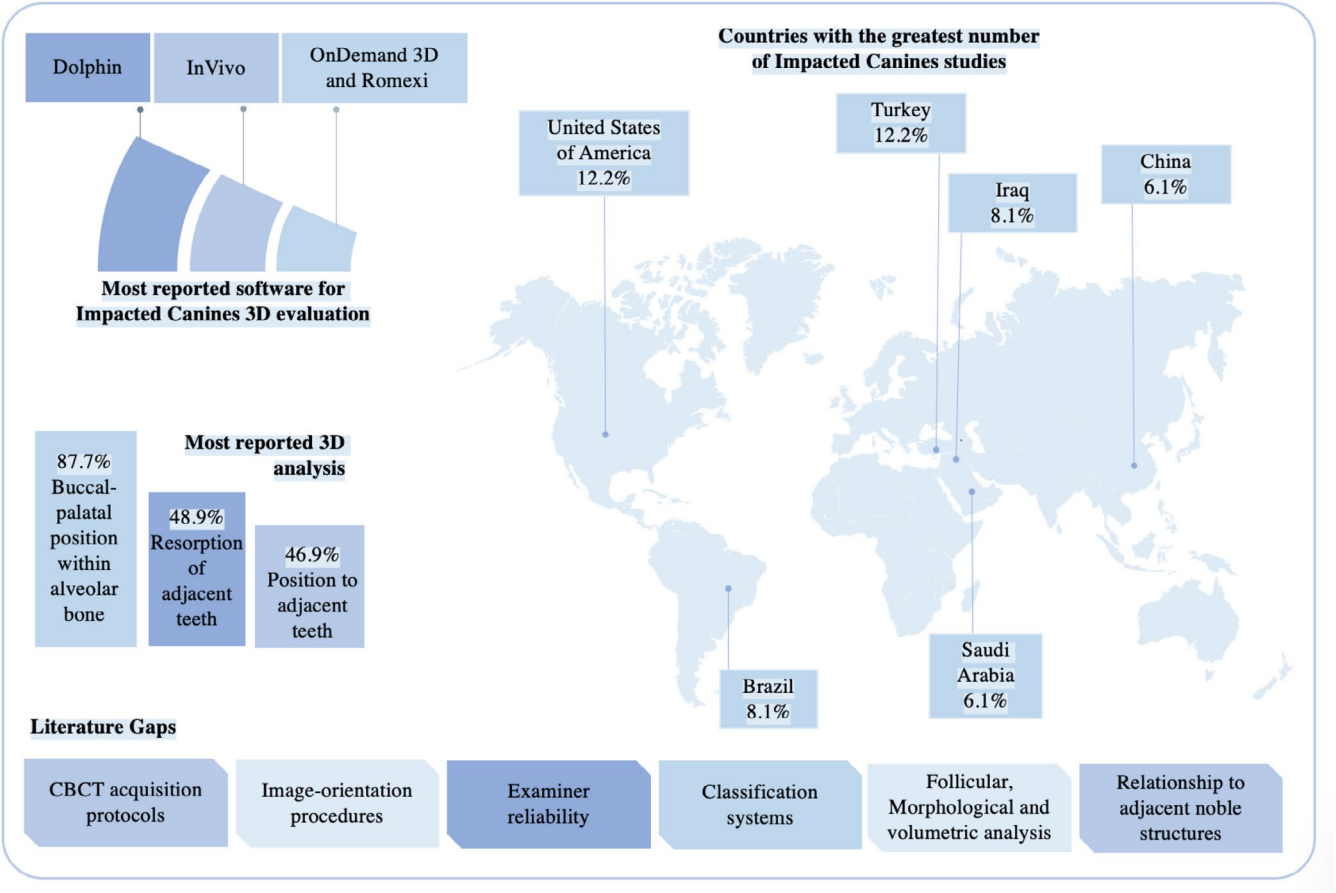
Mapping of methodological approaches used for the three‐dimensional evaluation of maxillary and mandibular.

From both clinical and research perspectives, the lack of standardized CBCT protocols identified in this review, particularly inconsistent reporting of voxel size, field of view, orientation procedures, and examiner calibration, may directly influence diagnostic interpretation, treatment planning, and risk assessment of impacted canines. Variability in acquisition parameters may affect the detection of early root resorption, while the absence of reproducible image reorientation may compromise the reliability of linear and angular measurements used to estimate eruption path and treatment difficulty. In light of these findings, greater attention to standardized acquisition settings, reproducible orientation protocols, and calibrated measurement approaches may enhance diagnostic consistency and improve interdisciplinary communication between orthodontists and radiologists.

In addition, the methodological heterogeneity identified in this review has implications beyond inter‐study comparability, particularly for the development and validation of quantitative and AI‐assisted diagnostic tools in impacted canine assessment. Machine learning and radiomics approaches depend on standardized acquisition parameters, reproducible orientation protocols, and consistent measurement frameworks to ensure feature stability and model reliability. Variability in voxel size, field of view, and segmentation workflows may reduce the reproducibility and generalizability of predictive models aimed at estimating root resorption risk, treatment difficulty, or eruption trajectory.

The predominantly descriptive nature of this synthesis reflects the methodological purpose of a scoping review, which aims to map the breadth and characteristics of existing evidence rather than conduct a formal risk‐of‐bias assessment or quantitative synthesis, in accordance with PRISMA‐ScR guidance. Nevertheless, structured narrative appraisal identified recurrent methodological constraints, including the predominance of retrospective cross‐sectional designs, inconsistent reporting of CBCT acquisition parameters, absence of standardized orientation protocols, lack of validated measurement frameworks, and limited examiner calibration [10, 69]. These factors may reduce reproducibility and inter‐study comparability. By systematically organizing these recurring domains, this review not only enhances interpretative transparency but also delineates specific methodological areas where refinement may strengthen future research and clinical reliability.

## 5 Conclusion

This scoping review mapped the methodological landscape of CBCT‐based three‐dimensional assessment of impacted canines and identified substantial variability in acquisition parameters, orientation protocols, measurement frameworks, and classification systems. Most investigations focused on maxillary canines, particularly evaluating bucco‐palatal position and adjacent root resorption, whereas mandibular impactions and standardized imaging workflows remain comparatively underexplored. Inconsistent reporting of CBCT methodologies limits reproducibility and generalizability. Greater harmonization of acquisition and evaluation protocols may enhance diagnostic consistency, support more reliable orthodontic treatment planning, and strengthen the integration of quantitative and AI‐assisted clinical approaches in impacted canine management.

## Author Contributions

Conceptualization: All authors. Design: All authors. Protocol preparation: All authors. Protocol revision and editing: All authors.

## Funding

The authors have nothing to report.

## Conflicts of Interest

The authors declare no conflicts of interest.

## Supporting information


**Table S1:** Data bases, search strategy and algorithms.

## Data Availability

The data supporting the findings of this scoping review are available from the corresponding author upon reasonable request.
