# Supplementary material for: Methodological Design for Three‐Dimensional Assessment of Maxillary and Mandibular Impacted Canines by Cone‐Beam Computed Tomography: A Scoping Review
Source: Orthod Craniofac Res. 2026 Jul 2;29(6):1067–93. doi: 10.1111/ocr.70154 (PMC13752646; doi:10.1111/ocr.70154)
Supplement: Supplementary file 1 [file OCR-29-1067-s001.docx]

Supplementary Table 01: Data Bases, Search Strategy and Algorithms.

| **Data Base** | **Search Strategy** | N |
| --- | --- | --- |
| **PubMed**  **(All Fields)** | Canine* OR "Impacted Canine" OR "Impacted Canines" OR "Ectopic Canine" OR "Ectopic Canines" OR "Unerupted Canine" OR "Unerupted Canines" OR "Retained Canine" OR "Retained Canines" | 105,823 |
|  | "Cone-Beam Computed Tomography" OR "Computed Tomography, Cone-Beam" OR "Cone Beam Computed Tomography" OR "CT Scan, Cone-Beam" OR "CT Scan, Cone Beam" OR "Cone-Beam CT Scan" OR "Cone-Beam CT Scans" OR "Scan, Cone-Beam CT" OR "Scans, Cone-Beam CT" OR "Tomography, Cone-Beam Computed" OR "Tomography, Cone Beam Computed" OR "Tomography, Volume Computed" OR "Computed Tomography, Volume" OR "Volume Computed Tomography" OR "Volumetric CT" OR "CT, Volumetric" OR "Volumetric Computed Tomography" OR "Computed Tomography, Volumetric" OR "Tomography, Volumetric Computed" OR "CAT Scan, Cone-Beam" OR "CAT Scan, Cone Beam" OR "CAT Scans, Cone-Beam" OR "Cone-Beam CAT Scan" OR "Cone-Beam CAT Scans" OR "Scan, Cone-Beam CAT" OR "Scans, Cone-Beam CAT" OR "Cone-Beam Computer-Assisted Tomography" OR "Computer-Assisted Tomography, Cone-Beam" OR "Cone Beam Computer Assisted Tomography" OR "Tomography, Cone-Beam Computer-Assisted" OR "Cone-Beam Computerized Tomography" OR "Computerized Tomography, Cone-Beam" OR "Cone Beam Computerized Tomography" OR "Tomography, Cone-Beam Computerized" OR "Cone-Beam CT" OR "CT, Cone-Beam" OR "Cone Beam CT" OR "Volume CT" OR "CT, Volume" OR "CBCT" | 34,330 |
|  | **#1 AND #2:**  ("canine*"[All Fields] OR "Impacted Canine"[All Fields] OR "Impacted Canines"[All Fields] OR "Ectopic Canine"[All Fields] OR "Ectopic Canines"[All Fields] OR "Unerupted Canine"[All Fields] OR "Unerupted Canines"[All Fields] OR "Retained Canine"[All Fields] OR "Retained Canines"[All Fields]) AND ("cone beam computed tomography"[MeSH Terms] OR "computed tomography cone beam"[All Fields] OR "cone beam computed tomography"[All Fields] OR "ct scan cone beam"[All Fields] OR "ct scan cone beam"[All Fields] OR "Cone-Beam CT Scan"[All Fields] OR "Cone-Beam CT Scans"[All Fields] OR "scan cone beam ct"[All Fields] OR "scans cone beam ct"[All Fields] OR "tomography cone beam computed"[All Fields] OR "tomography cone beam computed"[All Fields] OR "tomography volume computed"[All Fields] OR "computed tomography volume"[All Fields] OR "Volume Computed Tomography"[All Fields] OR "Volumetric CT"[All Fields] OR "ct volumetric"[All Fields] OR "Volumetric Computed Tomography"[All Fields] OR "computed tomography volumetric"[All Fields] OR ("cone beam computed tomography"[MeSH Terms] OR ("cone beam"[All Fields] AND "computed"[All Fields] AND "tomography"[All Fields]) OR "cone beam computed tomography"[All Fields] OR ("tomography"[All Fields] AND "volumetric"[All Fields] AND "computed"[All Fields])) OR "cat scan cone beam"[All Fields] OR "cat scan cone beam"[All Fields] OR ("cone beam computed tomography"[MeSH Terms] OR ("cone beam"[All Fields] AND "computed"[All Fields] AND "tomography"[All Fields]) OR "cone beam computed tomography"[All Fields] OR ("cat"[All Fields] AND "scans"[All Fields] AND "cone"[All Fields] AND "beam"[All Fields])) OR "Cone-Beam CAT Scan"[All Fields] OR "Cone-Beam CAT Scans"[All Fields] OR ("cone beam computed tomography"[MeSH Terms] OR ("cone beam"[All Fields] AND "computed"[All Fields] AND "tomography"[All Fields]) OR "cone beam computed tomography"[All Fields] OR ("scan"[All Fields] AND "cone"[All Fields] AND "beam"[All Fields] AND "cat"[All Fields])) OR ("cone beam computed tomography"[MeSH Terms] OR ("cone beam"[All Fields] AND "computed"[All Fields] AND "tomography"[All Fields]) OR "cone beam computed tomography"[All Fields] OR ("scans"[All Fields] AND "cone"[All Fields] AND "beam"[All Fields] AND "cat"[All Fields])) OR "cone beam computer assisted tomography"[All Fields] OR ("cone beam computed tomography"[MeSH Terms] OR ("cone beam"[All Fields] AND "computed"[All Fields] AND "tomography"[All Fields]) OR "cone beam computed tomography"[All Fields] OR ("computer"[All Fields] AND "assisted"[All Fields] AND "tomography"[All Fields] AND "cone"[All Fields] AND "beam"[All Fields])) OR "cone beam computer assisted tomography"[All Fields] OR ("cone beam computed tomography"[MeSH Terms] OR ("cone beam"[All Fields] AND "computed"[All Fields] AND "tomography"[All Fields]) OR "cone beam computed tomography"[All Fields] OR ("tomography"[All Fields] AND "cone"[All Fields] AND "beam"[All Fields] AND "computer"[All Fields] AND "assisted"[All Fields])) OR "cone beam computerized tomography"[All Fields] OR "computerized tomography cone beam"[All Fields] OR "cone beam computerized tomography"[All Fields] OR "tomography cone beam computerized"[All Fields] OR "cone beam ct"[All Fields] OR "ct cone beam"[All Fields] OR "cone beam ct"[All Fields] OR "Volume CT"[All Fields] OR "ct volume"[All Fields] OR "CBCT"[All Fields]) | 1187 |
| **Embase (ti,ab,kw)** | (Canine* OR "Impacted Canine" OR "Impacted Canines" OR "Ectopic Canine" OR "Ectopic Canines" OR "Unerupted Canine" OR "Unerupted Canines" OR "Retained Canine" OR "Retained Canines"):ti,ab,kw AND (("Cone-Beam Computed Tomography" OR "Computed Tomography, Cone-Beam" OR "Cone Beam Computed Tomography" OR "CT Scan, Cone-Beam" OR "CT Scan, Cone Beam" OR "Cone-Beam CT Scan" OR "Cone-Beam CT Scans" OR "Scan, Cone-Beam CT" OR "Scans, Cone-Beam CT" OR "Tomography, Cone-Beam Computed" OR "Tomography, Cone Beam Computed" OR "Tomography, Volume Computed" OR "Computed Tomography, Volume" OR "Volume Computed Tomography" OR "Volumetric CT" OR "CT, Volumetric" OR "Volumetric Computed Tomography" OR "Computed Tomography, Volumetric" OR "Tomography, Volumetric Computed" OR "CAT Scan, Cone-Beam" OR "CAT Scan, Cone Beam" OR "CAT Scans, Cone-Beam" OR "Cone-Beam CAT Scan" OR "Cone-Beam CAT Scans" OR "Scan, Cone-Beam CAT" OR "Scans, Cone-Beam CAT" OR "Cone-Beam Computer-Assisted Tomography" OR "Computer-Assisted Tomography, Cone-Beam" OR "Cone Beam Computer Assisted Tomography" OR "Tomography, Cone-Beam Computer-Assisted" OR "Cone-Beam Computerized Tomography" OR "Computerized Tomography, Cone-Beam" OR "Cone Beam Computerized Tomography" OR "Tomography, Cone-Beam Computerized" OR "Cone-Beam CT" OR "CT, Cone-Beam" OR "Cone Beam CT" OR "Volume CT" OR "CT, Volume" OR "CBCT"):ti,ab,kw) | 1095 |
| **Web of Science  (All Fields)** | **#1 ALL=(Canine* OR "Impacted Canine" OR "Impacted Canines" OR "Ectopic Canine" OR "Ectopic Canines" OR "Unerupted Canine" OR "Unerupted Canines" OR "Retained Canine" OR "Retained Canines")**  **#2 ALL=("Cone-Beam Computed Tomography" OR "Computed Tomography, Cone-Beam" OR "Cone Beam Computed Tomography" OR "CT Scan, Cone-Beam" OR "CT Scan, Cone Beam" OR "Cone-Beam CT Scan" OR "Cone-Beam CT Scans" OR "Scan, Cone-Beam CT" OR "Scans, Cone-Beam CT" OR "Tomography, Cone-Beam Computed" OR "Tomography, Cone Beam Computed" OR "Tomography, Volume Computed" OR "Computed Tomography, Volume" OR "Volume Computed Tomography" OR "Volumetric CT" OR "CT, Volumetric" OR "Volumetric Computed Tomography" OR "Computed Tomography, Volumetric" OR "Tomography, Volumetric Computed" OR "CAT Scan, Cone-Beam" OR "CAT Scan, Cone Beam" OR "CAT Scans, Cone-Beam" OR "Cone-Beam CAT Scan" OR "Cone-Beam CAT Scans" OR "Scan, Cone-Beam CAT" OR "Scans, Cone-Beam CAT" OR "Cone-Beam Computer-Assisted Tomography" OR "Computer-Assisted Tomography, Cone-Beam" OR "Cone Beam Computer Assisted Tomography" OR "Tomography, Cone-Beam Computer-Assisted" OR "Cone-Beam Computerized Tomography" OR "Computerized Tomography, Cone-Beam" OR "Cone Beam Computerized Tomography" OR "Tomography, Cone-Beam Computerized" OR "Cone-Beam CT" OR "CT, Cone-Beam" OR "Cone Beam CT" OR "Volume CT" OR "CT, Volume" OR "CBCT")**  **#1 AND #2: 1045 AND 29163** | 1045 |
| **SCOPUS**  **(All fields)** | TITLE-ABS-KEY ( canine* OR "Impacted Canine" OR "Impacted Canines" OR "Ectopic Canine" OR "Ectopic Canines" OR "Unerupted Canine" OR "Unerupted Canines" OR "Retained Canine" OR "Retained Canines" ) AND TITLE-ABS-KEY ( "Cone-Beam Computed Tomography" OR "Computed Tomography, Cone-Beam" OR "Cone Beam Computed Tomography" OR "CT Scan, Cone-Beam" OR "CT Scan, Cone Beam" OR "Cone-Beam CT Scan" OR "Cone-Beam CT Scans" OR "Scan, Cone-Beam CT" OR "Scans, Cone-Beam CT" OR "Tomography, Cone-Beam Computed" OR "Tomography, Cone Beam Computed" OR "Tomography, Volume Computed" OR "Computed Tomography, Volume" OR "Volume Computed Tomography" OR "Volumetric CT" OR "CT, Volumetric" OR "Volumetric Computed Tomography" OR "Computed Tomography, Volumetric" OR "Tomography, Volumetric Computed" OR "CAT Scan, Cone-Beam" OR "CAT Scan, Cone Beam" OR "CAT Scans, Cone-Beam" OR "Cone-Beam CAT Scan" OR "Cone-Beam CAT Scans" OR "Scan, Cone-Beam CAT" OR "Scans, Cone-Beam CAT" OR "Cone-Beam Computer-Assisted Tomography" OR "Computer-Assisted Tomography, Cone-Beam" OR "Cone Beam Computer Assisted Tomography" OR "Tomography, Cone-Beam Computer-Assisted" OR "Cone-Beam Computerized Tomography" OR "Computerized Tomography, Cone-Beam" OR "Cone Beam Computerized Tomography" OR "Tomography, Cone-Beam Computerized" OR "Cone-Beam CT" OR "CT, Cone-Beam" OR "Cone Beam CT" OR "Volume CT" OR "CT, Volume" OR "CBCT" )  TITLE-ABS-KEY ( canine* OR "Impacted Canine" OR "Impacted Canines" OR "Ectopic Canine" OR "Ectopic Canines" OR "Unerupted Canine" OR "Unerupted Canines" OR "Retained Canine" OR "Retained Canines" ) AND TITLE-ABS-KEY ( "Cone-Beam Computed Tomography" OR "Computed Tomography, Cone-Beam" OR "Cone Beam Computed Tomography" OR "CT Scan, Cone-Beam" OR "CT Scan, Cone Beam" OR "Cone-Beam CT Scan" OR "Cone-Beam CT Scans" OR "Scan, Cone-Beam CT" OR "Scans, Cone-Beam CT" OR "Tomography, Cone-Beam Computed" OR "Tomography, Cone Beam Computed" OR "Tomography, Volume Computed" OR "Computed Tomography, Volume" OR "Volume Computed Tomography" OR "Volumetric CT" OR "CT, Volumetric" OR "Volumetric Computed Tomography" OR "Computed Tomography, Volumetric" OR "Tomography, Volumetric Computed" OR "CAT Scan, Cone-Beam" OR "CAT Scan, Cone Beam" OR "CAT Scans, Cone-Beam" OR "Cone-Beam CAT Scan" OR "Cone-Beam CAT Scans" OR "Scan, Cone-Beam CAT" OR "Scans, Cone-Beam CAT" OR "Cone-Beam Computer-Assisted Tomography" OR "Computer-Assisted Tomography, Cone-Beam" OR "Cone Beam Computer Assisted Tomography" OR "Tomography, Cone-Beam Computer-Assisted" OR "Cone-Beam Computerized Tomography" OR "Computerized Tomography, Cone-Beam" OR "Cone Beam Computerized Tomography" OR "Tomography, Cone-Beam Computerized" OR "Cone-Beam CT" OR "CT, Cone-Beam" OR "Cone Beam CT" OR "Volume CT" OR "CT, Volume" OR "CBCT" ) | 1642 |
| **LIVIVO**  **(Open search)** | Canine* OR "Impacted Canine" OR "Impacted Canines" OR "Ectopic Canine" OR "Ectopic Canines" OR "Unerupted Canine" OR "Unerupted Canines" OR "Retained Canine" OR "Retained Canines" in Title Abstract Keyword AND "Cone-Beam Computed Tomography" OR "Computed Tomography, Cone-Beam" OR "Cone Beam Computed Tomography" OR "CT Scan, Cone-Beam" OR "CT Scan, Cone Beam" OR "Cone-Beam CT Scan" OR "Cone-Beam CT Scans" OR "Scan, Cone-Beam CT" OR "Scans, Cone-Beam CT" OR "Tomography, Cone-Beam Computed" OR "Tomography, Cone Beam Computed" OR "Tomography, Volume Computed" OR "Computed Tomography, Volume" OR "Volume Computed Tomography" OR "Volumetric CT" OR "CT, Volumetric" OR "Volumetric Computed Tomography" OR "Computed Tomography, Volumetric" OR "Tomography, Volumetric Computed" OR "CAT Scan, Cone-Beam" OR "CAT Scan, Cone Beam" OR "CAT Scans, Cone-Beam" OR "Cone-Beam CAT Scan" OR "Cone-Beam CAT Scans" OR "Scan, Cone-Beam CAT" OR "Scans, Cone-Beam CAT" OR "Cone-Beam Computer-Assisted Tomography" OR "Computer-Assisted Tomography, Cone-Beam" OR "Cone Beam Computer Assisted Tomography" OR "Tomography, Cone-Beam Computer-Assisted" OR "Cone-Beam Computerized Tomography" OR "Computerized Tomography, Cone-Beam" OR "Cone Beam Computerized Tomography" OR "Tomography, Cone-Beam Computerized" OR "Cone-Beam CT" OR "CT, Cone-Beam" OR "Cone Beam CT" OR "Volume CT" OR "CT, Volume" OR "CBCT" in Title Abstract Keyword - (Word variations have been searched) | 205 |
| **COCHRANE**  **(Title Abstract Keyword)** | Canine* OR "Impacted Canine" OR "Impacted Canines" OR "Ectopic Canine" OR "Ectopic Canines" OR "Unerupted Canine" OR "Unerupted Canines" OR "Retained Canine" OR "Retained Canines" in Title Abstract Keyword AND "Cone-Beam Computed Tomography" OR "Computed Tomography, Cone-Beam" OR "Cone Beam Computed Tomography" OR "CT Scan, Cone-Beam" OR "CT Scan, Cone Beam" OR "Cone-Beam CT Scan" OR "Cone-Beam CT Scans" OR "Scan, Cone-Beam CT" OR "Scans, Cone-Beam CT" OR "Tomography, Cone-Beam Computed" OR "Tomography, Cone Beam Computed" OR "Tomography, Volume Computed" OR "Computed Tomography, Volume" OR "Volume Computed Tomography" OR "Volumetric CT" OR "CT, Volumetric" OR "Volumetric Computed Tomography" OR "Computed Tomography, Volumetric" OR "Tomography, Volumetric Computed" OR "CAT Scan, Cone-Beam" OR "CAT Scan, Cone Beam" OR "CAT Scans, Cone-Beam" OR "Cone-Beam CAT Scan" OR "Cone-Beam CAT Scans" OR "Scan, Cone-Beam CAT" OR "Scans, Cone-Beam CAT" OR "Cone-Beam Computer-Assisted Tomography" OR "Computer-Assisted Tomography, Cone-Beam" OR "Cone Beam Computer Assisted Tomography" OR "Tomography, Cone-Beam Computer-Assisted" OR "Cone-Beam Computerized Tomography" OR "Computerized Tomography, Cone-Beam" OR "Cone Beam Computerized Tomography" OR "Tomography, Cone-Beam Computerized" OR "Cone-Beam CT" OR "CT, Cone-Beam" OR "Cone Beam CT" OR "Volume CT" OR "CT, Volume" OR "CBCT" in Title Abstract Keyword - (Word variations have been searched) | 153 |
| **LILACS**  **(Title, Abstract, Subject)** | (Canine* OR "Impacted Canine" OR "Impacted Canines" OR "Ectopic Canine" OR "Ectopic Canines" OR "Unerupted Canine" OR "Unerupted Canines" OR "Retained Canine" OR "Retained Canines") AND ("Cone-Beam Computed Tomography" OR "Computed Tomography, Cone-Beam" OR "Cone Beam Computed Tomography" OR "CT Scan, Cone-Beam" OR "CT Scan, Cone Beam" OR "Cone-Beam CT Scan" OR "Cone-Beam CT Scans" OR "Scan, Cone-Beam CT" OR "Scans, Cone-Beam CT" OR "Tomography, Cone-Beam Computed" OR "Tomography, Cone Beam Computed" OR "Tomography, Volume Computed" OR "Computed Tomography, Volume" OR "Volume Computed Tomography" OR "Volumetric CT" OR "CT, Volumetric" OR "Volumetric Computed Tomography" OR "Computed Tomography, Volumetric" OR "Tomography, Volumetric Computed" OR "CAT Scan, Cone-Beam" OR "CAT Scan, Cone Beam" OR "CAT Scans, Cone-Beam" OR "Cone-Beam CAT Scan" OR "Cone-Beam CAT Scans" OR "Scan, Cone-Beam CAT" OR "Scans, Cone-Beam CAT" OR "Cone-Beam Computer-Assisted Tomography" OR "Computer-Assisted Tomography, Cone-Beam" OR "Cone Beam Computer Assisted Tomography" OR "Tomography, Cone-Beam Computer-Assisted" OR "Cone-Beam Computerized Tomography" OR "Computerized Tomography, Cone-Beam" OR "Cone Beam Computerized Tomography" OR "Tomography, Cone-Beam Computerized" OR "Cone-Beam CT" OR "CT, Cone-Beam" OR "Cone Beam CT" OR "Volume CT" OR "CT, Volume" OR "CBCT") | 252 |
| **Google Scholar** | **Concept 1:** Impacted Canines  Canine OR "Impacted Canine" OR "Impacted Canines" OR "Ectopic Canine" OR "Ectopic Canines" OR "Unerupted Canine" OR "Unerupted Canines" OR "Retained Canine" OR "Retained Canines" OR Canino OR "Canino impactado" OR "Caninos impactados" OR "Canino ectópico" OR "Caninos ectópicos" OR "Canino no erupcionado" OR "Caninos no erupcionados" OR "Canino retenido" OR "Caninos retenidos" OR Canino OR "Canino Impactado" OR "Caninos Impactados" OR "Canino Ectópico" OR "Caninos Ectópicos" OR "Canino Não Erupcionado" OR "Caninos Não Erupcionados" OR "Canino Retido" OR "Caninos Retidos" AND | 229 |
|  | "Cone-Beam Computed Tomography" OR "Computed Tomography, Cone-Beam" OR "Cone-Beam CT Scan" OR OR "Volume Computed Tomography" OR "Tomography, Cone-Beam Computer-Assisted" OR "CBCT" OR “Tomografía Computarizada de Haz Cónico OR "Tomografía Computarizada, Haz Cónico" OR "Escáner CT de Haz Cónico" OR OR "Tomografía Computarizada Volumétrica" OR "Tomografía Computarizada Asistida por Computadora, Haz Cónico" OR "CBCT" OR Tomografia Computadorizada de Feixe Cônico OR "Tomografia Computadorizada, Feixe Cônico" OR "Escaneamento CBCT" OR OR "Tomografia Computadorizada Volumétrica" OR "Tomografia Computadorizada Assistida por Computador, Feixe Cônico" OR "CBCT" |  |
